# First person – Gabriel Matos-Rodrigues

**DOI:** 10.1242/dmm.047423

**Published:** 2020-10-30

## Abstract

First Person is a series of interviews with the first authors of a selection of papers published in Disease Models & Mechanisms, helping early-career researchers promote themselves alongside their papers. Gabriel Matos-Rodrigues is first author on ‘Progenitor death drives retinal dysplasia and neuronal degeneration in a mouse model of ATRIP-Seckel syndrome’, published in DMM. Gabriel conducted the research described in this article while a PhD student in Rodrigo A. P. Martins's lab at Universidade Federal do Rio de Janeiro, Rio de Janeiro, Brazil. He is now a PhD student in the lab of Bernard S. Lopez at Université de Paris, Paris, France, investigating mechanisms of DNA repair and DNA damage response.


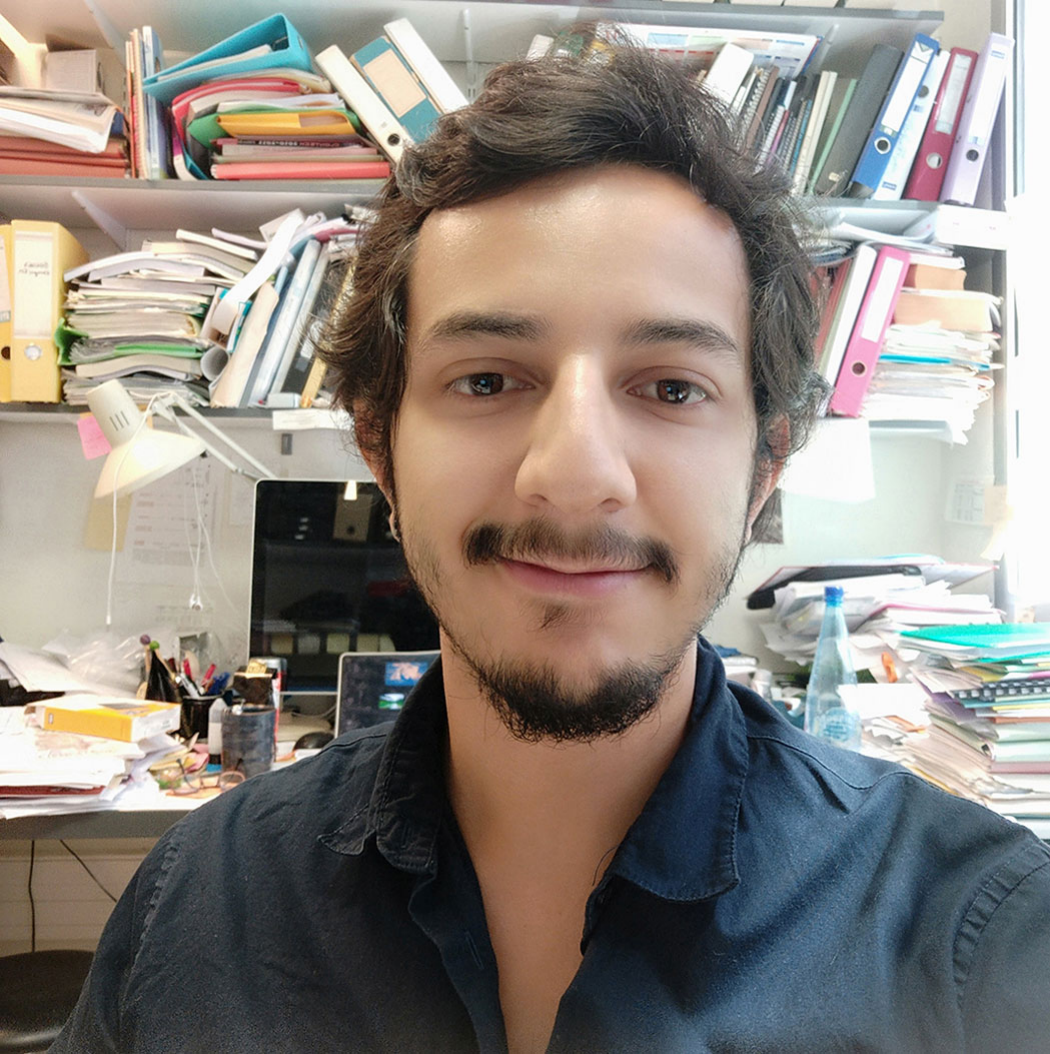


**Gabriel Matos-Rodrigues**

**How would you explain the main findings of your paper to non-scientific family and friends?**

Understanding how diseases that affect human development occur has been a particular challenge in biology. Seckel syndrome is a type of microcephalic primordial dwarfism (MPD) that is characterized by growth retardation and neurodevelopmental defects, including reports of retinopathy. When a proliferating cell, also referred to as a progenitor cell, duplicates its DNA before cell division, a molecular machinery called replication stress response (RSR) performs multiple quality control checks to guarantee that the genome is perfectly duplicated. Mutations in genes necessary for RSR, including the *Atrip* gene, are among the causes of Seckel syndrome. In our study, we asked how the retinal developmental defects observed in Seckel syndrome patients are formed. To do so, we developed mouse models to delete the *Atrip* gene specifically in the developing retina. We discovered that the ATRIP-Seckel syndrome mice were blind due to neurodegeneration of photoreceptor neurons, the cells that detect light within the retinal tissue. Proper retinal development relies on the precise coordination of the proliferation, differentiation and migration of retinal progenitor cells. We found that *Atrip*-deficient progenitor cells die in embryonic development, leading to defects in retinal tissue polarity, postnatal neurodegeneration and blindness. These data have important implications for the etiology of ocular manifestations observed in Seckel syndrome and other MPD patients.

**What are the potential implications of these results for your field of research?**

The findings reported in our paper are in agreement with a previously established model called ‘intrauterine programming’, used to explain the origins of developmental issues observed in patients of multiple human syndromes. According to this model, our findings show that deregulated RSR in embryonic retinal progenitor cells is the underlying cause of the severe visual deficits in adulthood. In addition, our results reveal a causal relationship between a cellular mechanism (progenitor cell death caused by defective RSR) and nervous system dysfunction (vision loss). Overall, our results in a mouse model of ATRIP-Seckel syndrome demonstrate that the death of progenitor cells may contribute to retinal malformations in Seckel syndrome and other MPD disorders.

**What are main advantages and drawbacks of the model system you have used as it relates to the disease you are investigating?**

The mouse has been an important ally of the scientific community for understanding human diseases. This is not only due to the close genetic relationship between mice and humans, but also because in many tissues, including the retina, human and mouse share the main developmental characteristics. In particular, transgenic mice are powerful tools and have been extensively used for investigations on the origins of genetic diseases. In our study, we developed a new mouse model that abolishes *Atrip* gene function specifically in the retina. One of the limitations of this approach is that the *ATRIP* mutation previously described in a Seckel syndrome patient was shown to reduce ATRIP protein levels, but not abolish it. Therefore, one of the limitations of our model is that it may not fully recapitulate the mutation found in the ATRIP-Seckel syndrome patient, because subtle differences in the amount of ATRIP protein in our model compared to the ATRIP mutant described in the Seckel syndrome patients may occur.

“[…] experimental biology is full of surprises.”

**What has surprised you the most while conducting your research?**

The most surprising issue while working in this project was to learn how well-thought hypotheses may end-up not being true. One of the first results that I obtained in this project was that retinal photoreceptor cells degenerate in ATRIP-Seckel syndrome mice. Based on previous studies, including clinical observations, we hypothesized that ATRIP may have a function in retinal photoreceptor neurons. It took us many years to understand that this hypothesis was not true, and to show that the underlying mechanism behind postnatal degeneration of photoreceptors was the death of retinal progenitor cells during embryonic development. I will always remember this experience as an example of how experimental biology is full of surprises.

**Describe what you think is the most significant challenge impacting your research at this time and how will this be addressed over the next 10 years?**

One of the biggest challenges in this field is the lack of patient-based data associated with genetic profiling. Because Seckel syndrome is a rare disorder, we still do not know the frequency of retinopathy in these patients or its relationship with specific disease-causing mutations. Genotype-phenotype associations focusing on the ophthalmological manifestations will be important contributions to the field. In addition, follow-up studies using primary tissue and retinal organoids from patient-derived induced pluripotent stem cells will be important to confirm the contribution of our observations in mice to the etiology of the retinal malformations.

**Figure:**
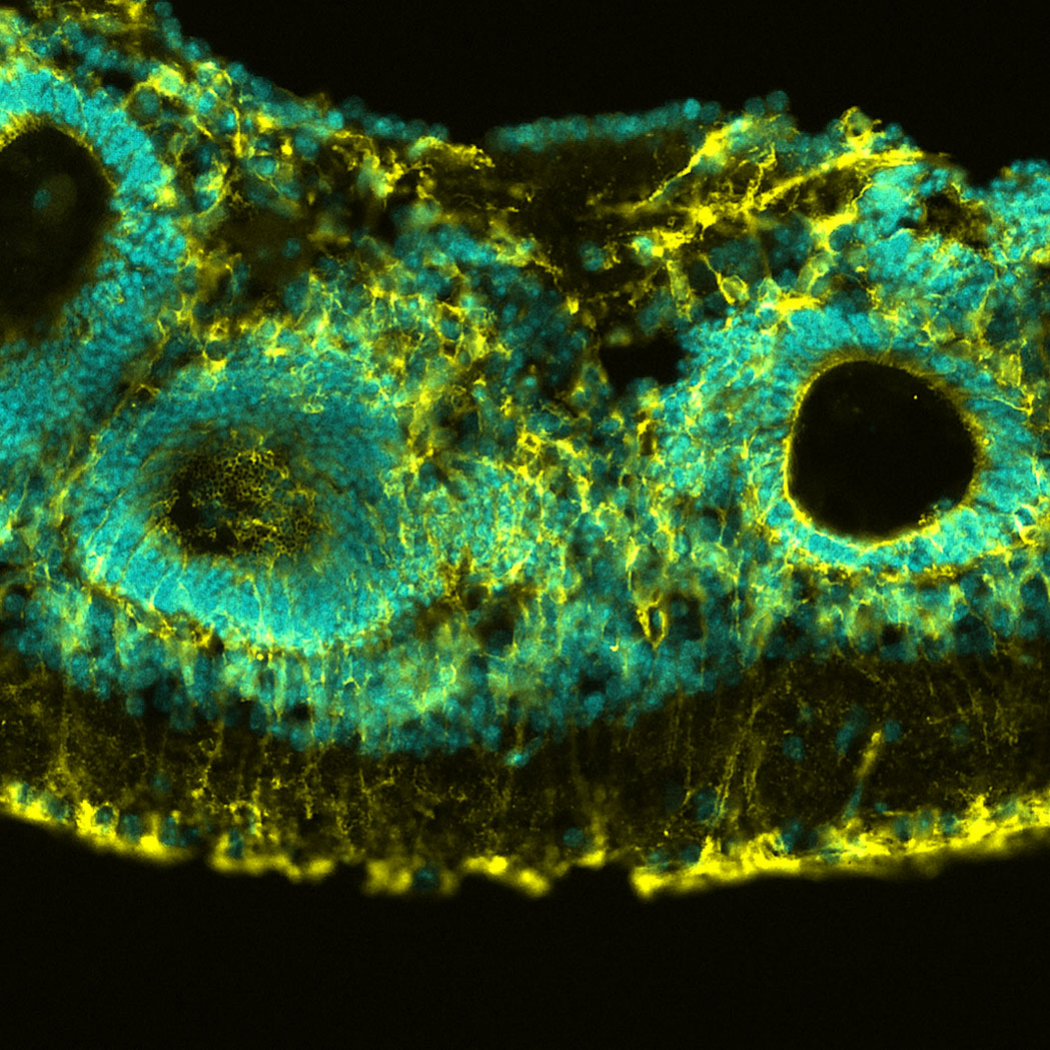
**Immunofluorescence illustrates tissue disorganization in the mature ATRIP-Seckel syndrome retina.**

“I am now witnessing the best minds of my generation in Brazil starving for opportunity.”

**What changes do you think could improve the professional lives of early-career scientists?**

As a young scholar in Brazil, my interest in science was propelled by social and political investment in research. In the past years, the system that allowed me to develop as a young scientist has been dismantled by lack of funding and disregard for scientific knowledge. I am now witnessing the best minds of my generation in Brazil starving for opportunity. Early-career scientists, including PhD students and postdocs, are a major driving force for scientific production in biology. I believe that improving work conditions has a great impact on the lives of early-career scientists. That includes access to funding, health care and vacations. Such measures may directly impact mental health, an ever growing issue in academia worldwide.

**What's next for you?**

During the thesis, my interest in the control of genomic stability was solidified. After my PhD defense, I plan to continue working on this topic, investigating the molecular mechanisms of DNA damage response and repair, as a postdoc in the group of Andre Nussenzweig at the NCI-NIH.
